# Hybrid Imaging in Industrial Applications: A Review of Principles and Deployment

**DOI:** 10.3390/jimaging12070309

**Published:** 2026-07-08

**Authors:** Andrzej Burghardt, Piotr Garbacz, Magdalena Muszyńska

**Affiliations:** Department of Applied Mechanics and Robotics, Faculty of Mechanical Engineering and Aeronautics, Rzeszow University of Technology, 35-029 Rzeszów, Poland; d583@stud.prz.edu.pl (P.G.); magdaw@prz.edu.pl (M.M.)

**Keywords:** hybrid imaging, quality inspection, image fusion

## Abstract

Hybrid imaging methods are emerging as one of the most dynamically evolving research areas in industrial inspection systems. This paper presents a literature review covering relevant scientific publications and official reports on the use of multimodal approaches in quality inspection and NDT systems. Hybrid imaging involves combining two or more imaging techniques to enhance the detection, characterization, and interpretation of features in inspected objects. The paper describes the physical foundations of vision-based inspection systems, including the interaction of optical radiation with matter. It also introduces a classification of optical methods and discusses the role of image fusion in multimodal data processing, with particular emphasis on high-speed quality control systems. The review outlines the current capabilities, limitations, and industrial applications of hybrid imaging, as well as future research directions, including integration with real-time systems and the use of artificial intelligence for automated defect interpretation.

## 1. Introduction

**Figure 1:**
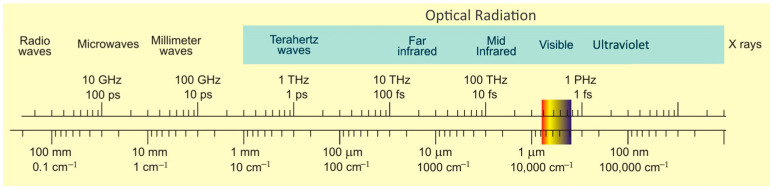
Electromagnetic spectrum. Adapted from [2].

Quality inspection and technical diagnostics of industrial equipment and machinery are rapidly evolving research areas. In modern manufacturing, 100% production inspection is gaining increasing importance. This is particularly relevant today, as one of the primary priorities of manufacturers is the implementation of sustainable production strategies aimed at minimizing waste and maximizing resource efficiency. An example of such initiatives is the Zero Defect Zero Waste (ZDZW) methodology [1], which seeks to achieve zero defects and zero waste in manufacturing, with non-destructive inspection methods serving as one of its fundamental pillars. One of the most extensively exploited physical phenomena in non-destructive inspection technologies (NDT) is the interaction between electromagnetic radiation and matter, which provides a basis for assessing the surface, subsurface, and structural integrity of inspected components. Across the broad range of techniques that utilize different portions of the electromagnetic spectrum (Figure 1), optical radiation imaging methods occupy a particularly prominent position.

The significance of optical imaging arises not only from rapid technological advancements in sensor architectures, optical components, illumination systems, and acquisition hardware, but also from ongoing progress in advanced image processing and computer vision algorithms that substantially improves detection sensitivity and measurement robustness. Additionally, optical techniques provide inherent scalability, supporting both high-resolution, close-range inspection and standoff measurements at considerable distances from the object. This versatility, together with the non-contact nature and suitability for high-throughput industrial environments, establishes optical imaging as a foundational element of non-destructive testing and a vital component of modern quality inspection systems.

The majority of current inspection methods rely on a single measurement technique. Ongoing research focuses on developing next-generation systems that offer enhanced automation, accuracy, and reliability. Hybrid or multimodal imaging combines different measurement techniques to support advanced image fusion approaches. Hybrid inspection systems provide significant advantages by capturing a wide range of data through images acquired from multiple sensor types. This methodology ensures redundancy and provides complementary information on potential defects in manufacturing processes. Selecting appropriate imaging techniques is essential, as each sensor modality possesses distinct limitations and characteristics. Once images of adequate quality are obtained, the subsequent step involves fusing data from these sources to achieve effective integration. Achieving the required level of effectiveness and reliability of an inspection system depends on the proper selection of technical and functional parameters determined by the aforementioned factors. Despite a significant increase in publications on image fusion methods in recent years, the design of imaging systems for hybrid optical inspection in industrial applications remains a persistent challenge [3].

## 2. Related Work

In this section, studies relevant to the scope of this review are introduced. Specifically, the development of multimodal medical and biological imaging methods is outlined as a pioneering field of application. In addition, hybrid solutions in military, surveillance, and remote sensing are summarized. Finally, several outstanding works in the area of structural health monitoring are presented.

### 2.1. Medical and Biological Hybrid Imaging

Numerous survey articles have delved into multimodal imaging in the field of medical and biological imaging. The first concepts of image fusion for hybrid nuclear imaging systems emerged at the turn of the 1990s, with early proposals for SPECT/CT and PET/SPECT modalities appearing in that period. Practical prototypes, however, were developed only several years later, when the initial ideas matured into implementable system designs [4,5]. In the following years, significant research has been undertaken to integrate molecular and optical imaging and other combinations, such as optical/MRI and optical–ultrasound systems. Over more than 25 years of development, hybrid imaging systems have provided an effective solution to overcome the limitations of individual modalities. In this context, three fundamental reasons for employing multimodal imaging in medicine were identified, which can be generalized as follows [6]:

- Acquiring complementary information;
- Achieving synergistic effects through data fusion;
- Planning future procedures and monitoring the actions taken.

Azhari et al. highlighted the relationship between accurate spatial alignment and the overall effectiveness of multimodal fusion. Authors noted that hardware-based solutions tend to be more efficient than post hoc alignment of independently acquired datasets using dedicated algorithms. However, these conclusions were formulated at a time when hybrid scanners were only beginning to enter clinical practice and before the advent of modern AI-based fusion methods. With the growing number of emerging concepts, Bischof et al. proposed categorizing these methods into two groups [7]: direct correlative and indirect. Hybrid methods are described as hardware platforms capable of simultaneously fusing two or more imaging techniques or sequentially to capture complementary information about the same sample and region of interest. Indirect multimodal imaging is used to study the same sample type but not necessarily at the same time or region. Post-acquisition processing is needed for integration between the recorded data. Moreover, the authors point out that in an ideal case, the use of hybrid methods in one device would avoid colocation of instruments issues, but due to the technical specifications of the imaging methods, combining them into single platforms is difficult. In a recent review, Diwakar et al. [8] emphasized that, despite ongoing efforts to apply advanced image-processing techniques based on deep-learning methodologies to mitigate misregistration artifacts, substantial progress is still required to attain the desired levels of accuracy and robustness. Moreover, Bhosekar et al. [9] highlight several limitations of CNN-based multimodal image fusion, including limited interpretability and high computational complexity, which constraint its applicability in real-time systems. Furthermore, biomedical imaging has traditionally prioritized spatial resolution while giving insufficient consideration to time as a critical factor in decision-making based on multimodal data [10].

### 2.2. Surveillance, Security and Military Applications

Hybrid imaging is widely used in surveillance and security systems [11] to integrate information from multiple cameras, thereby enhancing the appearance of observed objects—especially under low-light conditions. Typical applications include object recognition, tracking, anomaly detection, perimeter security, and situational awareness. Paramanandham, N., and Rajendiran, K. [12] presented a comparative study of image fusion techniques for surveillance applications. A large body of research focuses on fusing infrared and visible images to obtain a more informative result that is more suitable for the system operator.

In a recent study, Abdul-Al et al. [13] presented a comprehensive analysis of a hybrid multimodal biometric system (MBS) that integrates visible and infrared data to overcome the limitations of unimodal approaches and improve robustness, accuracy, and reliability.

In military applications, hybrid imaging systems incorporate both hardware and software solutions. In night-vision devices designed for soldiers, enhanced NVDs (dual-sensor night-vision devices). One promising approach involves monocular goggles that combine two optical channels—a digital night-vision channel and a thermal-imaging channel [14]. Dobrzycki A. and Bernardos A. propose a systematic framework for optimizing multimodal fusion in UAV object-detection systems [15]. In their paper, the authors highlight several key requirements that must be addressed in such applications:

- Modularity and fault tolerance—ensuring each modality can be processed independently;
- Interpretability—tracking back individual modalities for accountability and system validation;
- Computational efficiency—supporting real-time processing;
- Flexibility—allowing integration of new modalities or sensors.

Furthermore, the authors emphasize the importance of analyzing the trade-off between model complexity and performance, as well as implementing adaptive fusion mechanisms that balance precision and recall in real-time operation.

### 2.3. Hybrid Remote Sensing

Remote sensing is primarily used to obtain detailed geographic information through satellite platforms and UAV-based imaging systems. Earth observation and geomatics engineering rely on data from multimodal imaging systems to capture detailed characteristics of a wide variety of objects. To fulfill this demanding task, disparate modalities such as optical, SAR, LIDAR, and hyperspectral data are commonly employed. Samadzadegan et al. [16] analyzed a large number of studies and categorized the applications into the following domains: geophysical exploration, object recognition, thermal mapping, spatial, spectral and temporal resolution enhancement, agricultural applications, Land Use/Land Cover (LULC) mapping, and urban built-up mapping. However, with such advanced systems, several challenges must be addressed, including differences in spatial resolution, spectral domains, radiometric inconsistencies, and acquisition times.

Hybrid remote sensing systems require sophisticated software solutions. Classical remote sensing models based on machine learning and neural networks are typically designed for specific tasks and single data modalities, which limits their adaptability and versatility. Recently, Vision Transformer architectures and self-supervised learning have emerged as the most prominent approaches.

### 2.4. Structural Health Monitoring

Hybrid imaging has also been adopted in systems designed for Structural Health Monitoring (SHM) [17]. Ensuring the safety and integrity of infrastructures and complex engineered systems necessitates robust methods for the detection and characterization of degradation. One of the solutions is to employ multimodal image-fusion strategies, which enhance the reliability and completeness of diagnostic assessments. The optimal selection of one or more appropriate SHM methods necessitates the knowledge of the structural properties that are most significantly influenced by the damages of interest. Furthermore, SHM methods are prone to changing environmental influences and show different sensing capability and sensitivity to structural properties and structural changes [18]. Long-term lifecycle performance over years must be considered when designing systems dedicated to SHM. Employing a multi-sensor approach can provide a more comprehensive, accurate, and reliable assessment of structural damage.

However, these solutions do not account for the rigorous requirements of industrial environments. In most cases, the purpose of current fusion methods is to improve image quality solely to enable proper interpretation by the system operator, usually without considering the computational efficiency of the algorithms used [19].

## 3. Fundamentals of Vision-Based Inspection Systems

Traditional vision systems typically employ panchromatic imaging, where the camera is equipped with a single sensor tuned to a specific spectral range to capture the intensity of the averaged signal. Multispectral imaging (MSI) similarly utilizes one vision channel with a single sensor spanning a broad portion of the electromagnetic spectrum which is additionally equipped with a special mechanical or tunable filter system [20]. This approach makes it possible to capture images of the same scene across multiple wavelengths of electromagnetic radiation. Systems that leverage hyperspectral imaging (HSI) represent a further advancement of this technique, which enable the recording of several hundred images in various ranges of the electromagnetic spectrum [21]. Hyperspectral cameras are compact, integrated systems that generate a hyperspectral cube based on captured images within a given spectral range, maintaining a common frame of reference thanks to the manufacturer’s hardware and software solutions. A limitation of these systems is the difficulty in developing high-sensitivity sensors and optical elements with high transmittance across a wide range of electromagnetic radiation. To overcome this issue, hybrid systems may provide a solution. However, achieving high detection system efficiency requires a solid understanding of the physical principles underlying each imaging modality.

**Figure 2:**
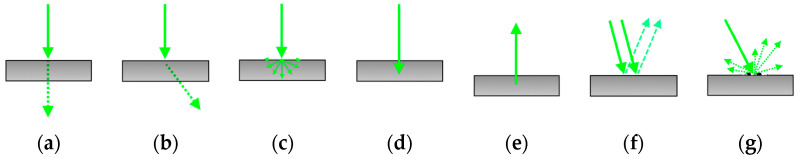
Types of EM radiation interactions: (**a**) transmission, (**b**) refraction, (**c**) diffusion, (**d**) absorption, (**e**) emission, (**f**) directional reflection, (**g**) diffuse reflection.

The way electromagnetic radiation affects any material can be used to analyze it qualitatively or quantitatively. A variety of interactions between optical radiation and matter (Figure 2) are harnessed to visualize the physical properties of objects. In practice, these phenomena rarely occur independently, most recorded images arise from the combined effects of interactions [22].

Depending on the wavelength employed and the properties of the object under investigation, particular interaction mechanisms may exert either a predominant or a minimal influence on the imaging process. The optical radiation spectrum consists of four principal bands: ultraviolet, visible, infrared, and terahertz. These regions can be further subdivided into segments, each characterized by distinct interaction properties.

### 3.1. Ultraviolet Radiation

The ultraviolet (UV) region of the electromagnetic spectrum comprises wavelengths shorter than those of visible light, spanning approximately 400 to 10 nm. A substantial portion of the ultraviolet band is non-ionizing. Due to various factors, the ultraviolet radiation range has been divided into smaller regions. Numerous conventions have been established for subdividing the ultraviolet spectrum into distinct subregions; however, an approach that is particularly well-suited to technical processes involves [23]:

- XUV—Extreme Ultraviolet (10–121 nm).
- FUV—Far Ultraviolet (122–200 nm).
- MUV—Middle Ultraviolet (200–300 nm).
- NUV—Near Ultraviolet (300–400 nm).

**Figure 3:**
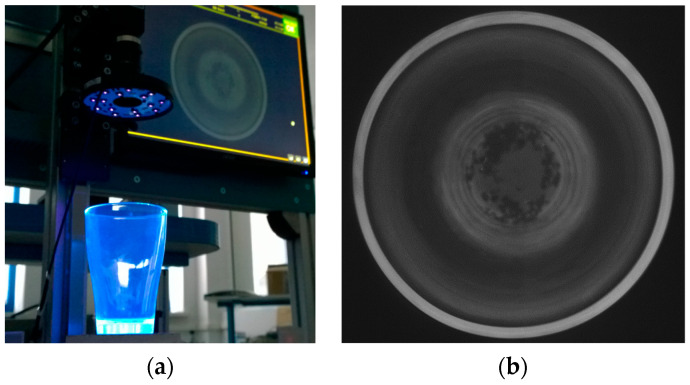
Fluorescence imaging system: (**a**) overview and (**b**) image recorded with VIS bandpass filter [24].

In electronics surface quality inspection applications, the term “deep UV” is often used, which ranges from 250 to 115 nm. Vacuum UV radiation (vacuum UV) is also used, covering wavelengths shorter than 180 nm. Due to substantial absorption of ultraviolet (UV) radiation by air, systems operating at wavelengths in this portion of the UV band often require a vacuum or an alternative atmosphere with higher transmissivity. The application of UV radiation in vision systems facilitates the visualization of features and properties of objects that are challenging to image using other modalities. Numerous substances exhibit pronounced absorption of UV radiation, permitting, among other benefits, the visualization of surface topography in materials that are transparent or semi-transparent to visible light. Moreover, the shorter wavelength of UV radiation results in significantly greater scattering compared to visible or infrared radiation when interacting with uneven surfaces. This property enables the detection of minute scratches or cracks that remain undetectable under other illumination conditions. One of the most commonly used features of ultraviolet radiation is the property of certain materials that allows the effect of fluorescence to be obtained (Figure 3) [24].

In methods based on this phenomenon, the object under investigation is illuminated with UV radiation, after which a visible-wavelength camera captures the photon emission from excited atoms or molecules. In systems employing active ultraviolet illumination and UV sensor, reflectance imaging is the predominant technique utilized [25].

### 3.2. Visible Light

Vision-based diagnostic and quality control systems typically use visible radiation, also referred to as visible light. The electromagnetic spectrum corresponding to this type of radiation closely matches the sensitivity range of the human eye, encompassing wavelengths from approximately 380 to 780 nm. Visible radiation was first used in imaging systems and is now widely used in industrial vision systems. A special feature of the visible spectrum is the ability to identify colors. Quality control systems that rely on color analysis require specially engineered sensors. The preferred method for acquiring color images uses a photosensitive array with a Bayer filter. In this approach, light incident on the camera sensor passes through a mosaic of red, green, and blue optical filters [26]. Another solution for capturing color images is a triple imager. These cameras use three image sensors with filters for red, green, and blue, along with a prism [27]. Moreover, within the visible spectrum, numerous methods have been developed for 3D inspection of products in manufacturing systems [28]. These methods demonstrate high precision and accuracy in assessing manufactured components, thereby supporting the stringent quality-control requirements characteristic of demanding sectors such as the aviation industry [29].

In general, non-contact measurement techniques that utilize the visible spectrum are predominantly employed in inspection processes such as [30]:

- Inspection of shape and geometric dimensions;
- Position recognition;
- Completeness checking;
- Surface inspection;
- Comparison of objects and images;
- Object identification (using codes and tags).

**Figure 4:**
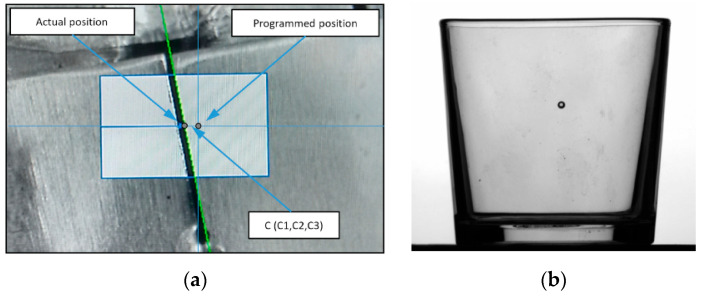
Imaging systems include: (**a**) reflective systems—welding path identification [31], and (**b**) transmission systems—glassware inspection [33].

In the design of industrial vision systems, the primary objective is to enhance the contrast of features of interest in the observed object while minimizing contrast in non-relevant areas of the acquired images. The appropriate design of imaging systems plays a critical role in ensuring the reliability and repeatability of vision systems. Reflectance imaging remains the principal technique for acquiring images in the visible spectrum. It is used in geometric measurements and the detection of surface defects in products or positioning task in robotics applications (Figure 4a) [31]. In a transmission imaging system, the object under inspection is illuminated from behind, toward the camera, which records the transmitted radiation. This configuration is referred to as backlighting. For opaque objects, this method is commonly employed to measure the external dimensions of a product or checking the completeness of complex products [32].

During the inspection of transparent materials, the light transmitted through the object’s structure and captured by the camera sensor is analyzed. This approach enables the detection of subsurface defects, including internal structural damage and inclusions (Figure 4b) [33].

### 3.3. Infrared Radiation

It is assumed that the infrared radiation spectrum (IR) encompasses a band of wavelengths above 0.78 µm. The upper limit of this band, depending on the source, can reach 1000 µm, a characteristic of older publications that do not include the now-separated terahertz band. Owing to the sensitivity of materials utilized in the fabrication of camera sensors, as well as the presence of atmospheric windows with high transmissivity, the infrared range is divided into sub-ranges.

- NIR—near-infrared (0.78–1 µm).
- SWIR—short-wave infrared (1–3 µm).
- MWIR—middle-wave infrared (3–5 µm).
- LWIR—long-wave infrared (8–14 µm).
- VLWIR—very-long-wave infrared (14–1000 µm).

The majority of industrial inspection systems utilize commercially available cameras that operate within the NIR, SWIR, MWIR, and LWIR spectral ranges.

**Figure 5:**
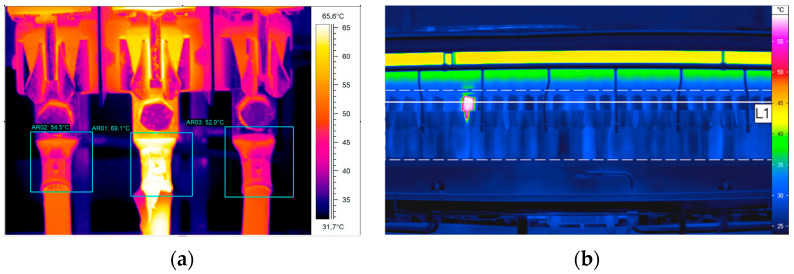
Thermal camera images: (**a**) indoor switchboard [36], (**b**) process of washing bottles [37].

Imaging methods utilizing infrared radiation can be broadly categorized based on the imaging of electromagnetic waves transmitted through, reflected from, or emitted by an object as a result of its thermodynamic properties. In practical applications, reflected infrared radiation primarily encompasses the short-wave infrared range. Similar to systems operating in the visible spectrum, illumination of the object is required to obtain an image in these systems. An example of leveraging these properties of NIR radiation is the imaging of transmitted infrared radiation and reflectography used in non-destructive testing of works of art [34]. In industrial contexts, the high absorption of many fluids in the SWIR band is frequently utilized. Imaging systems of this type are employed to inspect moisture content or to measure liquid levels [35]. Nevertheless, infrared radiation is primarily employed to image thermal processes occurring on surface of observed objects (Figure 5).

These systems utilize electromagnetic radiation from the mid-wave and long-wave infrared regions, collectively referred to as thermal infrared (TIR) [38]. The spectral distribution of blackbody emittance, as described by Wien’s displacement law, has significant practical implications for selecting appropriate sensors to measure the temperature of a given object [39]. Long-wave infrared (LWIR) cameras are engineered to measure temperatures within the range of −70 °C to 250 °C. In the short-wave infrared (SWIR) region, temperatures from 250 °C to 800 °C are typically measured. Higher temperatures may also be measured using CCD and CMOS sensors, which are generally intended for imaging in the visible spectrum [40]. Thermal imaging systems can be divided into passive and active solutions. Passive thermography refers to the measurement of thermal variations in materials using an infrared imaging device, without the requirement for external thermal stimulation. In contrast, active thermography requires the application of external heat sources. Depending on the stimulation method, several types of active thermography are distinguished [41]:

- Pulsed thermography;
- Stepped thermography;
- Lock-in thermography;
- Pulsed-phase thermography;
- Frequency-modulated thermography.

Pulse thermography involves determining and analyzing the temperature distribution on a tested surface as it cools after being stimulated with a thermal pulse of a specified duration, up to the point where the surface returns to its baseline temperature. Modulation (synchronous) thermography is also often referred to as lock-in thermography.

### 3.4. Terahertz Radiation

Terahertz radiation spans the electromagnetic spectrum from 0.1 to 10 THz, corresponding to wavelengths from 3 mm to 30 µm. The sub-terahertz band is often defined as the range from 0.1 to 1 THz. For many years, the field of optical radiation was not fully explored. This was due to difficulties in developing both sources and detectors capable of operating at THz frequencies. This band was often referred to as the terahertz gap [42]. The recent development of terahertz imaging methods is largely attributable to advances in technical solutions that enable the production of compact radiation sources and detectors operating within this region of the electromagnetic spectrum. Ongoing technological progress continues to broaden the range of applications for terahertz imaging systems. Nevertheless, the effective implementation of these systems necessitates a comprehensive understanding of both the inspection process and the physical properties of the inspected objects. Terahertz systems are transitioning from the laboratory research phase to industrial applications; however, their widespread adoption is still constrained by stringent requirements for reliability and resistance to interference. Terahertz waves are characterized by several unique properties:

- Low attenuation in dielectrics (textiles, paper, plastic, leather, wood);
- High absorption by polar substances (e.g., water);
- Complex and individual spectral signatures of materials;
- High reflectivity of metals.

**Figure 6:**
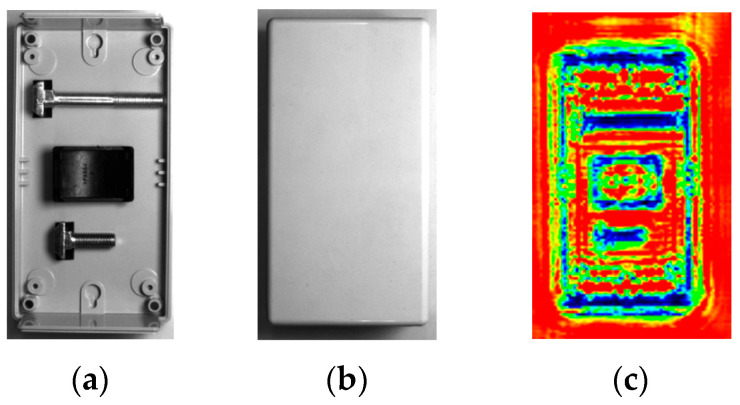
Enclosure (**a**) interior, (**b**) exterior, (**c**) sub-terahertz image [43].

These features allow terahertz imaging to penetrate objects and reveal their internal structure. For example, it enables the inspection of a package without opening it (Figure 6).

Terahertz radiation enables diverse applications, notably in public safety, defense, quality control, and technical diagnostics [44]. Although terahertz imaging offers numerous advantages, it is also subject to notable limitations, chiefly low imaging resolution and the prevalence of diffraction effects [45]. The penetration depth of terahertz radiation and the sensitivity of the inspection system are dependent on the source power. In the case of low-power sources, the use of technologically advanced sensors with enhanced sensitivity becomes essential. Despite progress in THz detector research, the number of commercially available solutions remains extremely limited. The main technological barriers hindering the wider application of this imaging method revolve around several key issues. First, the insufficient spatial resolution of currently available THz sensors is a fundamental limitation, further exacerbated by their high cost, which is directly dependent on the number of measurement elements in the sensor. A significant challenge is the lack of dedicated optical systems, which necessitates the use of lensless systems, whose field of view and observation capabilities are limited by the sensor size. Finally, the performance parameters of THz cameras—such as low pixel counts and restricted frequency ranges—remain insufficient to meet the stringent requirements of industrial environments.

## 4. Hybrid Imaging Inspection Methods

**Figure 7:**
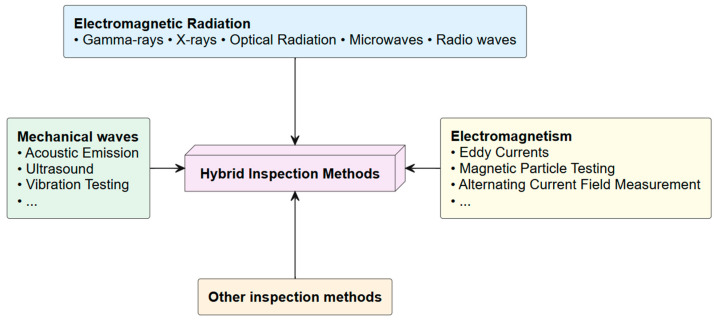
Generic hybrid inspection methods.

In general, hybrid systems can combine the advantages of measurement methods using various physical phenomena such as penetrating radiation, optical radiation, acoustic waves, electromagnetism and other inspection methods (Figure 7).

Hybrid methods increase the probability of detecting specific features of the object being inspected and enable synergistic effects, yielding new information that is not possible with either technique used alone. To illustrate the range of applications and possible configurations, Table 1 presents selected examples of hybrid systems used in technical diagnostics and quality control. Many studies focus on composite inspection using hybrid techniques. Torbali et al. provided an extensive literature review of this method’s application to the structural inspection of composites, including those used in aviation [46]. Inspection systems employing hybrid approaches are being developed for a variety of sectors, including the food industry, electronics, art conservation, and safety applications. To provide a comprehensive and consistent comparison of the selected solutions, the table presents the achieved algorithmic performance. Furthermore, all systems were evaluated for their Technology Readiness Level (TRL) according to the NASA scale. The scope and primary purpose of the implemented data fusion approach are also indicated.

**Figure 8:**
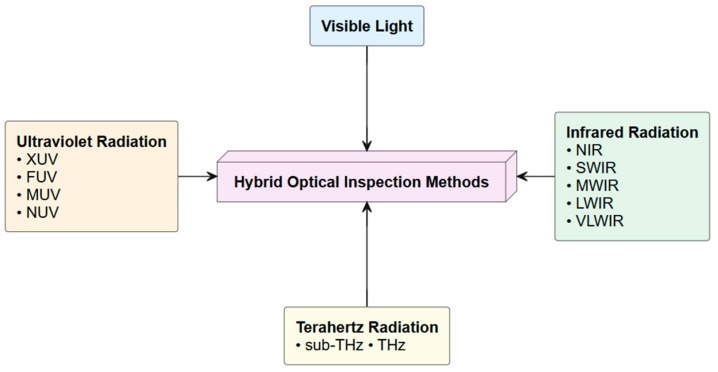
Hybrid optical inspection methods.

One of the most widely utilized physical phenomena in non-destructive testing is the interaction between electromagnetic radiation and matter, which enables the examination of surfaces, sub-surfaces, and internal structures of objects. Among the various methods that employ different regions of the electromagnetic spectrum, optical imaging techniques are particularly significant. This significance is attributable, in part, to the rapid advancement of sensors, optical systems, and image processing and analysis techniques. Furthermore, optical methods are characterized by high scalability and the ability to record radiation at various distances from the object under investigation. Hybrid approaches leveraging the optical radiation spectrum (Figure 8), due to the unique properties of electromagnetic waves in individual bands, facilitate the design of systems with enhanced detection efficiency and reliability.

The manner in which electromagnetic radiation interacts with matter, depending on the wavelength employed, is of particular significance, as are optical phenomena that directly influence measurement accuracy, including diffraction, distortion, and chromatic aberration [60]. The selection of appropriate optical radiation ranges to be used in a hybrid system depends on many factors, including:

- The test object (e.g., structure, material type, physical properties);
- Measurement requirements (e.g., frequency, resolution);
- Object characteristics (e.g., geometric dimensions, structure, temperature).

Based on a review of the current state of the art, a unified methodology for the design and implementation of hybrid vision systems has not been identified. Concurrently, the literature underscores the importance of appropriately selecting the most pertinent modalities, as these facilitate the analysis of factors contributing to quality variability in inspected products. In extensive research on subsurface imaging, Saha characterizes the design process as an iterative optimization task that begins with problem definition, followed by probe selection based on an evaluation of candidate solutions. This evaluation considers each probe’s sensitivity to relevant physical parameters and its ability to provide adequate spatial resolution. The author identifies the key optical parameters that must be considered when designing an inspection system: absorption, scattering, diffusion coefficient, refractive index, fluorescence rate and lifetime, and, for thermal imaging, thermal emittance. Subsequent steps include selecting an appropriate imaging configuration and testing the system on representative sample targets, typically preceded by simulation-based evaluation. Finally, end-to-end system optimization is performed using defined performance metrics to ensure that the system meets the required specifications. Saha explicitly notes that multimodal imaging should be employed when a single modality exhibits inherent limitations, such as insufficient contrast or inadequate resolution.

To complement this approach, the authors of this work suggest that the design of hybrid optomechatronic systems as inherently complex structures should incorporate decision-support methods when selecting among possible solutions. One viable method is the use of a multi-criteria decision matrix developed according to the Pugh concept selection method [61]. In the first stage of concept development, it is necessary to define the selection criteria and assign appropriate weights. The primary criteria should describe the system’s ability to detect specific classes of defects, such as surface defects, subsurface defects, or thermal anomalies. Additional criteria may relate to the quality of the acquired images: for example, those influenced by illumination intensity and uniformity across the inspection area, image blur, or spatial resolution. Subsequently, criteria that capture the analytical capabilities of the hybrid system as a whole should be included, such as the ability to perform defect classification or the synergistic benefits arising from combining multiple imaging modalities. Finally, practical implementation criteria must be considered. These include the technology readiness of individual imaging modules (e.g., IP rating, compliance with communication standards, and expected operational lifespan), operational requirements (e.g., the need for frequent calibration), system scalability, adaptability, expandability, and the cost of producing additional units.

In the developed decision matrix, it is essential to present the baseline variants that are the single-modality configurations as the first entries. These serve as benchmarks against which all subsequent hybrid configurations are evaluated. Depending on the nature of the project, whether research-oriented or implementation-oriented, the weights assigned to criteria related to TRL levels, cost, and practical deployment aspects should take on correspondingly higher values. In implementation projects, these factors typically dominate the decision process, whereas in model-oriented research studies, such criteria may be assigned lower weights, reflecting their reduced relevance at early conceptual stages. Ultimately, the development and implementation of hybrid systems that utilize the optical radiation spectrum require an interdisciplinary approach that considers the underlying physical phenomena in optical inspection systems, as well as methods for image fusion and data acquisition.

### 4.1. Image Fusion Methods

**Figure 9:**
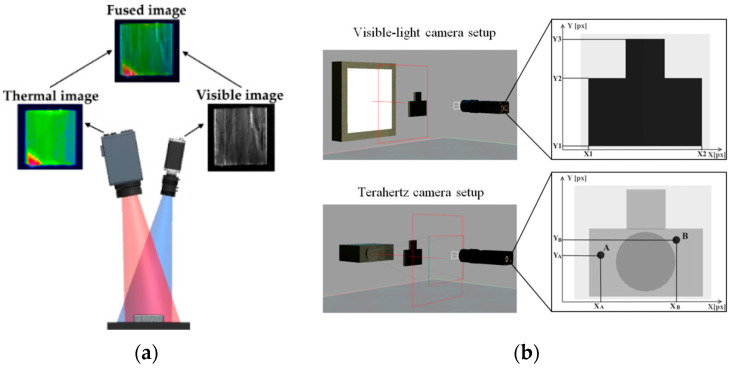
Image fusion concept in hybrid systems: (**a**) IR-VIS [62], (**b**) VIS-THz [42].

Image fusion (Figure 9a) is the process of integrating information from multiple images to enhance reliability and achieve greater overall usability than any individual input image in context of image quality regarding feature contrast and resolutions.

Image fusion represents a specific form of data fusion, the implementation of which enables [63]:

- Greater detail in information representation.
- Increased measurement accuracy.
- Elimination of interference and measurement errors.
- Comprehensive imaging of phenomena.

**Figure 10:**
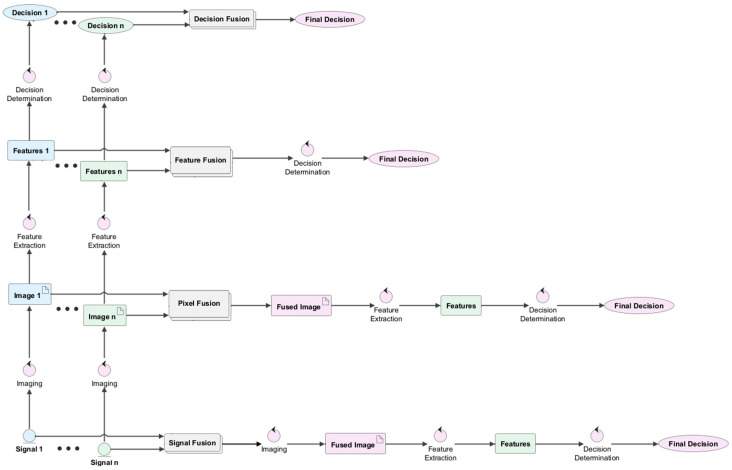
Fusion techniques at different processing levels: signal-level, pixel-level, feature-level, and decision-level fusion.

The fusion of data from multiple sources necessitates consideration of additional challenges associated with their processing and analysis—challenges that do not arise in systems employing a single measurement technique [64]: data incommensurability, resolution disparities, image registration, partial data absence or data conflict. Different acquisition techniques inherently generate not only diverse data types but also distinct categories of errors. When integrating information from multiple sources, the individual observations vary in their confidence, reliability, and overall quality. The presence of more than one information source inevitably introduces potential conflicts, discrepancies, and inconsistencies. Furthermore, calibrating multimodal imaging methods is more complex than calibrating unimodal solutions. A commonly used approach involves employing a calibration pattern with specific points that exhibit high contrast across all applied modalities [62]. However, for imaging methods based on fundamentally different physical principles, establishing geometric relationships is a particularly challenging task [47]. Integrating images with other data types (e.g., 2D images and time-series electrical signals) requires precise calibration and temporal coordination. In such hybrid systems, synchronization can be achieved by using a shared clock to trigger both acquisition systems [57]. Additionally, geometric registration may be performed using reference markers. Another issue is the choice of the processing stage at which image fusion [65] will be performed (Figure 10). Early fusion can diminish modality-specific features, whereas late fusion may not adequately capture cross-modal interactions. Identifying required sensor types enables more effective fusion, especially when combining features extracted from data across techniques, which is more advantageous than processing raw signals. However, when data types are completely incommensurable, the only feasible approach remains fusion of inspection results (decision fusion).

Image fusion methods are typically categorized into several main groups [66]:

- Multiscale transform methods [67].
- Sparse representation techniques [68].
- Deep-learning-based methods [69].
- Saliency-based approaches [70].
- Hybrid models integrate various approaches to enhance fusion quality.

With respect to the above methods, solutions that explicitly focus on fusion for local anomaly detection remain relatively rare. These systems employ local feature extraction and fusion strategies based on region segmentation or the isolation of specific regions of interest (region-based fusion). Such approaches enable the preservation of detailed information about key image elements, which in applications such as visual inspection, object recognition, and medical image analysis, provides a significant advantage over the traditional global approach.

Historically, the initial requirement for image fusion involved employing methods that efficiently and ergonomically combine signals from these sensors for presentation to a human operator [71]. However, the influence of image synthesis on task-specific analysis algorithms and their results is currently attracting attention. In the study by Singh and Tuwari, the impact of image fusion on classification is explored to determine the optimal combination for improving classification accuracy [72]. In a recent study, Alshohoumi and Al-Hamdani evaluated fusion-based models demonstrating that the choice of fusion strategy (early, intermediate, or late) substantially influences classification performance [73].

Relatively fewer publications on image fusion explicitly address the computational efficiency of the algorithms utilized. Nonetheless, Kalamkar and Mary [11] highlight the relationship between algorithmic complexity and processing time for various image modalities. Multimodal and hyperspectral fusion are the most complex and time-consuming. In contrast, bispectral fusion is characterized by low complexity and shorter computational times. For multimodal and multisensory fusion, both complexity and processing time can vary significantly depending on the number of modalities, sensor types, and algorithms employed. Wang et al. [74] highlight that spatial fusion methods, which operate through direct manipulation of pixel values, are distinguished by their low computational complexity and straightforward implementation. However, the effectiveness of these methods is highly contingent upon the accuracy of pixel weight estimation. In deep learning-based approaches, the use of larger models typically translates into improved representational and learning capabilities, but at the cost of increased computational and memory requirements. The number of model parameters often correlates with the number of floating-point operations (FLOPs), and increasing the number leads to longer processing times. Consequently, in practical real-time inspection systems implementations, these parameters should be analyzed collectively rather than in isolation. Ding et al. [75] recently demonstrated that robust multimodal image fusion critically depends on a reliable pre-registration stage, as early fusion pipelines require strict pixel-level alignment of corresponding physical locations. Most solutions based on dense networks and GAN-based approaches are limited in preserving complex, high-quality details and are susceptible to redundant structures and mode collapse. Consequently, recent fusion methods have become increasingly task-oriented. Building on these developments, the authors propose a Modality-Invariant Progressive Representation (MIPR) approach that achieves excellent accuracy results. However, the limitations of these methods become particularly apparent in inference benchmarks: for low-resolution input images (399 × 282 px), high accuracy is only achievable with extended inference times (~0.67 s). At higher resolutions, the trade-off between accuracy and latency becomes increasingly pronounced, underscoring the need for image registration techniques that are both computationally efficient and geometrically robust. General-purpose image registration and fusion methods can be used in less time-critical applications, but often struggle in demanding scenarios, particularly when multimodal data exhibit weakly discriminative or significantly different features. In online industrial inspection applications, which are typically tailored to specific tasks and permit precise system calibration, incorporating a priori knowledge of object properties and process variability enables the development of computationally efficient and robust image registration algorithms even for high-resolution imagery [76]. This approach accelerates the entire image fusion process and makes it more suitable for real-time applications.

### 4.2. Applications of Hybrid Optical Imaging in Industrial Inspection

The primary goal of hybrid inspection systems designed for industrial applications is the accurate assessment of an object’s condition based on captured images within a strictly defined and limited time frame. An important consideration in the design of such systems is the selection of appropriate sensors and lighting that ensure high-quality images, which is closely determined by the specific application of the given system. It depends not only on the type of material of the examined element, but also on the characteristic of the potential defects that need to be detected or classified. When designing hybrid quality control systems, the following factors must be taken into account:

- Characteristics of the sensors and optical systems used;
- Inspection zone illumination methods;
- Mechanical constraints;
- System immunity to interference factors.

Furthermore, in high-throughput manufacturing environments, such as the fast-moving consumer goods (FMCG) sector, quality control systems are frequently required to operate in real time to ensure comprehensive inspection and zero-defect production. In non-destructive testing methods, data analysis is typically conducted as a post-process activity. Consequently, in online applications, a large number of industrial systems still rely on classical machine vision solutions, whereas in offline scenarios, AI-based methods are used more frequently. This observation is consistent with recent comparative studies on rule-based systems and data-driven approaches in industrial monitoring [77]. Classical rule-based algorithms provide rapid and reliable responses, typically with low computational requirements. In contrast, data-driven methods exhibit high performance across many practical systems.

High-throughput manufacturing environments require rapid and reliable decision-making within the production cycle. For example, inspection rates in the tobacco industry can reach up to 10,000 articles per minute [78], strongly favoring classical algorithmic approaches. In comparison, glass tableware manufacturing typically operates at approximately 180 pieces per minute [24], making AI-based methods more practical. Consequently, designers of industrial inspection systems must carefully assess the feasibility of different algorithms in the context of application-specific throughput requirements. An extensive survey of semiconductor manufacturing systems [79] demonstrates that achieving millisecond-level inference often requires a trade-off between inspection speed and accuracy. Furthermore, a recent study by Jayawardena et al. [80] emphasizes the importance of reporting both average inference time and worst-case latency, as the latter directly affects system responsiveness and operational safety. Performance metrics such as frames per second (FPS) and inference time per image (ms/image) should therefore be reported consistently to facilitate meaningful comparisons among different methods.

These considerations become even more critical in hybrid inspection systems, where multimodal data acquisition increases both the amount of information to be processed and the computational burden associated with preprocessing and image registration. As a result, latency and computational constraints become increasingly pronounced. Consequently, field deployment often relies on simpler or compressed models, including those obtained through model compression, quantization, or knowledge distillation. Such approaches accept a modest reduction in performance in exchange for computational feasibility and real-time operation on resource-constrained hardware. Table 2 presents selected examples of hybrid optical inspection systems used in quality control and non-destructive testing across a variety of industrial applications.

Analysis of the selected systems demonstrates that employing a hybrid optical method which integrates modalities with significant properties, such as visible light and infrared imaging, improves inspection efficiency through redundancy and complementarity. The synergy effect refers to how combining information from different image sources enables more comprehensive detection, highlighting features that may be missed using a single modality [86]. However, this benefit is often not clearly stated or emphasized in the analyzed literature. Moreover, few authors explicitly address the importance of high throughput or discuss the necessity of properly designing hybrid vision systems to match the required inspection frequency [83]. Recent comprehensive waste-sorting datasets highlight the need for further research on image registration and fusion in high-speed applications [92]. As presented in the paper, such datasets should contain registered multimodal images and pixel-wise ground-truth annotations. In addition, all technical parameters of the acquisition process should be reported to ensure reproducibility. As a baseline, high-resolution visible-light images should be included alongside modalities that are relevant to the inspection objective. The authors should also specify the desired minimal processing time for registration and model inference and present the performance of their own solution as a benchmark result.

## 5. Discussion

**Figure 11:**
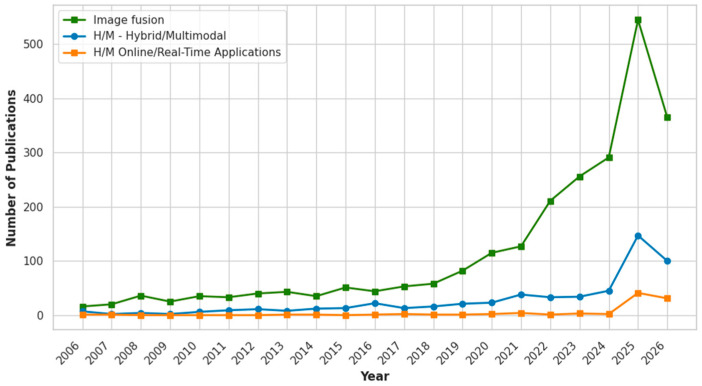
Annual distribution (up to June 2026) of relevant publications on hybrid/multimodal imaging and image fusion in industrial implementations.

To identify research trends in hybrid imaging and image fusion, a literature search was conducted using the Scopus database. The analysis focused on publications related to industrial inspection, quality control, and non-destructive testing (NDT). To ensure thematic relevance, the search was limited to subject areas closely associated with engineering and industrial applications, including Engineering, Computer Science, and Multidisciplinary Sciences. The queries were implemented using Scopus field restrictions, requiring the imaging-related terms to appear in the title or abstract and the inspection-related terms to appear in the title, abstract, or author keywords. For general hybrid/multimodal systems identification, the search formula was constructed as follows: (“hybrid imaging” OR “multimodal imaging”) AND (“inspection” OR “quality control” OR “NDT” OR “nondestructive testing” OR “non-destructive testing”). To broaden the coverage of relevant industrial applications, the strategy was refined by including publications indexed with selected Scopus keywords such as Quality Control, Inspection, Defect Detection, Defects, Nondestructive Examination, Computer Vision, Image Processing, Image Quality, Thermography, Infrared Imaging, Hyperspectral Imaging, Imaging Systems, Optical Data Processing, Multimodal, Adaptive Optics, Learning Systems, Real-Time Systems, and Quality Assurance. This approach was intended to comprehensively capture studies combining multiple imaging modalities and advanced image-processing techniques for industrial inspection and quality assurance applications. This filtering approach enabled the identification of publications specifically addressing imaging-based inspection applied in industrial environments. However, a substantial proportion of the screened studies focuses on hybrid concepts in AI methodologies, rather than on the implementation of multimodal imaging systems themselves. The body of literature, including publications up to June 2026, demonstrates a steady growth in research activity over the earlier years, followed by a pronounced surge within the last two years. Figure 11 highlights this recent acceleration in publication output. While most studies continue to concentrate primarily on image processing, an increasing number of multi-source and multi-data investigations reflects the growing scientific and industrial interest in hybrid and multimodal imaging techniques.

However, many of these studies did not present actual implementations of multimodal imaging systems; instead, the integration was frequently highlighted as a promising avenue for future research and development, reflecting a growing interest in the field. Current academic trends align with the conclusions reached by leaders in the industrial vision systems sector [93]. Experts identify the growing prominence of AI-based systems and multimodal sensing technologies as vital research areas for improving inspection reliability and minimizing misclassification risks in preventing process defects. As demonstrated in this review, online and real-time implementations remain limited. Despite a noticeable increase in the number of publications, significant challenges continue to hinder the translation of these approaches into robust industrial applications. This persistent gap underscores the critical need for further research directed at overcoming practical barriers.

When designing complex structures such as hybrid imaging architectures, decision-support methods should be employed to guide the selection of viable solutions. This includes a preliminary assessment of imaging modalities capable of meeting defined requirements, followed by a systematic comparison of alternative concepts against a baseline according to the established criteria. The implementation of a hybrid system necessitates both ex ante evaluation before deployment and ex post evaluation after a sustained period of operation. These evaluations are critical for ensuring traceability, reliability, and industrial applicability. It is worth noting the issue-appropriate metrics for evaluating fusion quality in industrial applications. To design an optimal configuration of a hybrid system, it is necessary to employ image-quality measures that objectively quantify how different variables influence detection performance. Existing methodologies include both subjective and objective approaches, and image-quality assessment is widely applied across various scientific and engineering fields. Most established metrics focus on perceptual aspects of visual quality and aim to predict image fidelity based on properties of the human visual system. Subjective evaluation methods rely on experts or observers who assess displayed images using a predefined rating scale. Conversely, objective methods employ mathematical models to automatically and quantitatively estimate image quality. Torbali et al. recommend using five metrics for overall image fusion evaluation: root mean square error (RMSE), signal-to-noise ratio (SNR), correlation coefficient (CC), fusion mutual information (MFI), and structural similarity index (SSIM) [46]. However, in industrial applications, implementing full-reference methods is often impractical because reference images are typically unavailable. In these situations, synthetic reference data generated by advanced rendering engines can be utilized. Alternatively, evaluation may be limited to no-reference metrics. In inspection system design, a common criterion is the maximization of contrast between a defect and its immediate surroundings. In many cases, Michelson contrast is used for this purpose. However, for defect-detection tasks, greater robustness to local disturbances is provided by the modified contrast, which computes the difference between the median pixel values of the defect region and its nearest neighborhood [26]. This measure enables the evaluation of images in terms of their potential effectiveness in subsequent image-analysis algorithms.

Similar to other inspection systems, a hybrid solution should facilitate the co-creation of value by generating actionable insights and recommendations that promote positive transformation within production environments. The overarching purpose of quality inspection is to enhance organizational performance and operational efficiency through the identification and elimination of process and product nonconformities, as well as the mitigation of their root causes. The primary goal of implementing hybrid systems is to achieve synergy between the utilized modalities. From a metrology perspective [94], this combined operation can enhance system performance through expanded spatial and temporal coverage and improved resolution. It can also provide greater robustness to sensor and algorithm uncertainty. Additionally, it enables more effective noise suppression and leads to higher measurement accuracy. The implementation of hybrid solutions for industrial applications should also be justified in terms of return on investment [95]. Although many studies focus primarily on performance improvements, fewer provide a detailed cost–benefit analysis, and in some cases, the additional complexity and expense of multimodal approaches may yield only modest gains compared with single-modality solutions. Furthermore, the inspection process should support continuous improvement by identifying effective practices and guiding the refinement of standards, procedures, and operating methods. A quality-control closed-loop concept can be implemented to enable online and continuous improvement of manufacturing processes [96]. Hybrid systems may serve as a robust foundation for implementing predictive quality [97], an advanced approach to data management and control that anticipates potential deviations prior to the occurrence of irregularities. Nevertheless, successful implementation necessitates addressing challenges associated with data incommensurability, such as difficulties in image registration, incomplete modality-specific data, and conflicts among heterogeneous data sources.

It is expected that Industry 4.0 elements, such as digital twins, hybrid systems, and artificial intelligence, will be widely applied while concurrently enhancing holistic industrial processes [98]. However, progress in these solutions depends heavily on standardized methodologies and the availability of representative datasets. Open or real-world industrial multimodal databases are essential for algorithmic development and benchmarking, yet remain limited. In parallel, existing synthetic data-generation and simulation environments must be extended to support non-visible modalities, enabling more realistic modeling of industrial conditions and sensor interactions [99]. Such developments would facilitate the validation of AI models on real-world, noisy data and support the optimization of model architectures for strict real-time requirements that are characteristic of in-line industrial inspection.

## 6. Conclusions

Reliable inspection is essential for achieving Zero Defect Zero Waste goal in modern manufacturing processes. Optical inspection techniques provide non-contact, rapid solutions by utilizing various bands of the electromagnetic (EM) spectrum. However, reliance on a single modality is at times insufficient to ensure reliable outcomes, which has stimulated increased attention toward multimodal methods. Hybrid inspection systems can deliver both complementary and redundant information, which is particularly valuable for detecting anomalies in industrial processes. Fusing data from diverse sources as redundancy enhances robustness and safety in contexts involving consumer health risks, such as pharmaceutical or food inspection applications. Additionally, the integration of complementary information offers expanded insight into inspected objects and processes and contributes to achieving operational-efficiency targets. In certain cases, integrating multiple modalities produces synergistic effects, resulting in performance improvements that surpass those attainable with individual data sources.

Although substantial progress has been made in leveraging the concept of hybrid imaging in fields such as medicine, surveillance, and defense, most existing image-based systems still rely on post-processing strategies that are inadequate for in-line industrial quality control and remain applicable only to NDT or other off-line inspection scenarios. Furthermore, the conducted study reveals a research gap in the methodology for designing and deploying hybrid solutions. The development process for such systems is typically carried out both sequentially and iteratively. At each phase, verification activities should be performed to advance the system to a higher TRL. If the defined requirements are not fulfilled, the workflow permits a return to earlier design alternatives to continue refinement. In such situations, the concept selection matrix prepared during the initial stage enables the designer to move to the next ranked option in accordance with the established evaluation hierarchy. Moreover, further efforts are needed to establish robust methods for assessing image-fusion quality in inspection tasks and to enhance the use of synthetic data as input for deep learning models dedicated to quality-control applications. A critical issue concerns the reliability and integrity of the data, which directly influences both the performance of fusion algorithms and the generalization capability of learning-based inspection systems.

In high-speed production environments, an important challenge arises from the requirement for real-time results, which necessitates balancing model-architecture complexity with processing efficiency. Employing optical, vision-based modalities—typically operating within similar regions of the electromagnetic spectrum, such as visible or near-infrared light—can simplify image registration compared with hybrid configurations that combine modalities based on fundamentally different physical principles (e.g., thermal imaging or X-ray). These cross-spectral combinations often introduce additional alignment difficulties due to variations in spatial resolution, contrast mechanisms, and sensor characteristics. While these optical modalities are information-rich, they can be difficult to interpret; from an automation perspective, the nature of the data remains a challenge [100]. A critical consideration in designing hybrid inspection solutions is to ensure that fusion strategies prioritize input data optimized for analytical algorithms, which may not always correspond to the way a human operator perceives the fused images.

In the era of Industry 4.0 and IoT solutions, data fusion has become an important research focus. Production automation is increasingly shifting toward predictive quality. Combining sensor data enables the development of more accurate and robust models that adapt to real operating conditions. Nevertheless, ensuring high-quality data for predictive models, as well as implementing reliable methods for detecting sensor faults, remains a critical challenge.

## Figures and Tables

**Table 1:** Selected hybrid inspection methods.

Utilized Technologies	Object of Inspection (Dataset Size)	Inspection Objective	Fusion Level	Fusion Objective	Mode	Throughput (Real-Time)	TRL
X-ray, Optical 3D [47]	Food Products: Poultry filets (20 samples)	Internal defects detection (Bone fragments)	Pixel Fusion	Enhanced contaminant detection through compensation for meat thickness-induced X-ray attenuation effects.	In situ	Overall system: 0.2 m/s (RT tested)	5
RGB, 3D, Hyperspectral, X-ray [48]	Food Products: Onions (74 samples)	Internal and external defects detection (decay, softness, sunscald, sprouting, mechanical damage), size/shape out of tolerance.	Feature Fusion	Improved defect detection accuracy (Hybrid—81.58%, X-ray only—73.68%, HSI only—78.95%) and complementary weight estimation	In situ	Individual acquisition: X-ray—0.24 m/s RGB—12 FPS 3D—30 FPS HIS—N/A (Non-RT tested)	5
Ultrasounds, Thermography, Eddy current [49]	CFRP composites (1 sample)	Internal and external defects detection	Pixel Fusion	Enhanced and improved detection traceability	Ex situ	Individual acquisition UT—0.3 m/s EC—0.3 m/s PT—N/A	4
Ultrasounds, Pulsed Thermography [50]	CFRP composites (3 samples)	Internal defects detection	Decision Fusion	Improved defect detection (Increased size of defect region in hybrid mode) and classification (impact characterization) Enhanced and improved detection traceability	Ex situ	N/A	4
Ultrasounds, X-ray CT [51]	CFRP composites (16 samples)	Internal defects detection	Pixel Fusion	Complementary characterization of internal damage geometry	Ex situ	N/A	4
Optical (3D, Shearography), Thermography, Laser Ultrasonic [52]	Aircraft components (2 samples)	Internal and external defects detection	Pixel Fusion	Complementary defect information. Improved defect classification	Ex situ	N/A	4
Eddy current, Ultrasounds, Microwaves [53]	Aircraft Lap-Joints (5 samples)	Internal and external defects detection	Decision Fusion	Increased accuracy of corrosion material loss (Actual—16 ÷ 17%, Hybrid—17.4%, EC—13%) and crack detection (Hybrid—98.8%, EC—88.9%)	Ex situ	N/A	4
Optical (LBR), Acoustic Emission, X-Ray [54]	Laser welding (200 samples)	Internal defects detection	Feature Fusion	Improved defect classification accuracy (2–3% increase with hybrid AE/LBR), X-ray for comprehensive information regarding defect formation	In situ	Overall system: 1.5 m/s (RT tested, comp. time—2 ms)	5
Induction Thermography, Eddy current [55]	Aluminum/carbon fiber composite (1 sample)	Internal defects detection	Feature Fusion	Complementarity of inspection (IT—core damage, delamination, EC nature and position of core defects)	Ex situ	N/A	4
Optical, Infrared, X-ray [56]	Paintings (2 samples)	Internal and external defects detection (cracks)	Feature Fusion	Increased defect detection efficiency	Ex situ	N/A	4
Optical, Ultrasounds [57]	Steel pipes (45K samples)	Internal and external defects detection	Feature Fusion	Complementarity of inspection. Improved defect detection accuracy (Hybrid—97.3%, VIS—90.8%, UT—92.5%)	In situ	Overall system: 35 ms/sample (RT tested)	5
Optical IR/VIS, Electrical [58]	Welded metal joints (884 samples in test set)	Internal and external defects detection (cracks, porosity)	Feature Fusion	Improved defects classification accuracy (Hybrid—89.5%, VIS < 70%, IR < 65%, EL < 75%)	In situ	Overall system: 0.25 m/s (RT tested)	5
Thermography, Acoustic [59]	Leak detection (168 samples in IR dataset. 84 samples in AE dataset)	External defects detection Small (<0.25 mm), Medium, Large (>1.0 mm) leaks	N/A	Optimized detection and localization	In situ	(RT tested)	3

Note: N/A stands for “not available” or “not applicable”.

**Table 2:** Selected hybrid optical inspection methods.

Utilized Technologies	Object of Inspection (Dataset Size)	Inspection Objective	Fusion Level	Fusion Objective	Mode	Throughput (Real Time)	TRL
Hyperspectral VIS-NIR + SWIR [81]	Food products: grape berries (200 samples)	Internal defects detection (soluble solids content)	Feature Fusion	Reduced Error of Prediction (Hybrid RMSEP = 0.65%, VIS-NIR RMSEP = 0.76%, SWIR RMSEP = 0.7%)	In situ	30 mm/s (RT focused)	4
UV/VIS/NIR [82]	Food products: cherry tomato (115 samples)	Internal defects detection (Lycopene content)	Feature Fusion	Improved prediction accuracy of lycopene (Hybrid—R^2^ = 0.95)	In situ	N/A (RT focused)	3
VIS/IR [62]	Food products: heat-sealing container (66 samples)	Internal and external defects detection (creases, wrinkles, contaminations, weak seals)	Pixel Fusion	Prediction of seal strength (Hybrid IR/VIS/Process data—R^2^ = 0.82, Hybrid IR/VIS—R^2^ = 0.42)	In situ	400 mm/s (RT tested)	4
UV/VIS [83]	Wood (3 types fresh, stored, dry)	External defects detection (Fungal infections)	Feature Fusion	Comprehensive information regarding defect formation Increased ability to detect co-occurring infections	In situ	N/A (RT focused)	4
VIS/IR [84]	PCB electronic boards	Internal and external defects detection (Epoxy overflow, die scratches, voids, bubbles)	Pixel Fusion	Enhanced precision (Hybrid—99.5% mAP@0.5) and robustness	In situ	Model —60 FPS VIS cam —19 FPS IR cam —50 FPS (RT focused)	4
Photoluminescence/VIS [85]	SiC wafers (188 samples in valid set)	Internal and external defects detection (Micropines, pits, bumps, inclusions)	Feature Fusion	Enhanced defect detection coverage and reliability (Hybrid—85% accuracy)	Ex situ	10 wafers per hour	8
VIS/IR [86]	Aluminum profiles	Internal and external defects detection (cracks, blisters, scratches, weld defects, die lines, streak defects)	Pixel Fusion	Comprehensive information on defect types (Prediction of streak defects)	In situ	Processing time—50 ms (RT focused)	4
VIS/IR [87]	GMA Welding (12 samples)	Process instabilities detection	Pixel Fusion	Improved classification accuracy (Hybrid—59%, VIS—51%, IR—39%)	In situ	32 cm/min (RT focused)	4
VIS/IR [88]	Friction stir welding	Internal and external defects detection (cracks, cavities, excessive burr)	Decision Fusion	Comprehensive information on defect types	In situ	10 mm/s (RT focused)	4
VIS/IR [89]	Turbine blade	External defects detection	Pixel Fusion	Detailed view, enhanced contrast	Ex situ	N/A (RT focused)	4
UV/IR [90]	Aircraft exhaust detection (5400 samples)	Nozzle type and speed classification	Pixel Fusion	Increased classification accuracy (Hybrid—97.48, IR—96.54, UV—86.76)	In situ	N/A (RT focused)	3
UV/IR [91]	Power grid insulation (10 samples)	Insulation faults detection	Pixel Fusion	Improved accuracy of fault diagnosis (Hybrid—94%)	In situ	N/A (RT focused)	4
RGB/SWIR/IR/THz [92]	Bulky waste (22,659 samples)	Materials sorting	Pixel Fusion	Robust classification (F1 score: Hybrid—93%, RGB—86%, NIR—89%, IR—79%, THz—64%)	In situ	N/A	5

Note: N/A stands for “not available” or “not applicable”.

## Data Availability

The original contributions presented in this study are included in the article. Further inquiries can be directed to the corresponding author.
